# Team Member Work Role Performance: The Organizational Benefits From Performance-Based Horizontal Pay Dispersion and Workplace Benign Envy

**DOI:** 10.3389/fpsyg.2020.566979

**Published:** 2020-12-03

**Authors:** Haiyan Zhang, Shuwei Sun, Lijing Zhao

**Affiliations:** ^1^Business School, Jiangsu Normal University, Xuzhou, China; ^2^School of Mathematics and Statistics, Xuzhou University of Technology, Xuzhou, China; ^3^Business School, Nanjing University, Nanjing, China

**Keywords:** performance-based HPD, team member work role performance, workplace benign envy, pay position, moderated mediation

## Abstract

In the context of the current uncertain, complex, and interdependent work systems, teams have become organizations’ substantial working unit, which in turn challenges the traditional view of employee performance and ultimately results in the emergence of team member work role performance. Employee team-oriented work role behaviors with proficiency, adaptivity, and proactivity, which are integrated by the new construct, are so crucial to team effectiveness that many organizations keenly expect to achieve team member work role performance through implementing a dispersed pay-for-performance plan within a team. This study seeks to address the organizational practitioners’ main concern that whether pay dispersion among team members (i.e., horizontal pay dispersion, HPD) could actually help realize team member work role performance and further examines why and when an employee could respond to HPD within a team by engaging in team member work role behaviors from the perspective of the performance-shaping basis and team member’s workplace benign envy. Drawing on emotion-related theory, social comparison theory, legitimacy theory, expectation theory, and relative deprivation theory, it proposes that performance-based HPD could not only positively impact team member work role performance via workplace benign envy but also exert a direct-positive effect. Moreover, the activating effect of performance-based HPD on workplace benign envy and the mediating role are much stronger when a team member’s pay position is higher. The multi-source data including objective information and subjective perception among 362 ordinary employees within 66 Chinese organizational teams primarily supported the moderated mediation model. Yet, the direct-positive effect was not established.

## Introduction

Over the past few decades, many organizations have adopted teams as their substantial working unit (Park et al., 2013) to cope with the greatly uncertain, complex, and interdependent work systems (Howard, 1995). This advances the idea that employee performance no longer depends on to what extent an employee performs his or her tasks and responsibilities, as an individual, prescribed in his or her static job description, but depends more on whether he or she and his or her coworkers could work well in a team member role. Put differently, the growing uncertainty, complexity, and interdependence within work systems and organizations’ widespread adoption of teams have challenged the traditional view of employee performance, as it could not fully account for employee team-oriented behaviors with proficiency, adaptivity, and proactivity as a team member in such a dynamic context where teams are in prevalent use (Murphy and Jackson, 1999). Therefore, Griffin et al. (2007) put forward a new construct *team member work role performance* to integrate *employee team-oriented behaviors with proficiency*, which mainly respond to the growing interdependence of work systems, and *employee team-oriented behaviors with adaptivity and proactivity*, which combine to cope with the increasing uncertainty and complexity of work systems. Obviously, team member work role performance is essentially a kind of employee behavioral performance by definition. Given that teams are in great prevalence and employee team-oriented behaviors are crucial to team effectiveness, many organizational practitioners are carrying out a dispersed pay-for-performance plan within a team with keen expectations to motivate employees to engage in team member work role performance.

It has always been an important practical and theoretical issue capturing widespread social concern and interest from practitioners and scholars on what kind of pay dispersion tends to encourage employees to behave or perform to their organizations’ expectations. The extant research has already yielded some significant insights about the relationship between pay dispersion and employee outcome performance (Becker and Huselid, 1992; Bloom, 1999; Aime et al., 2010; Uriesi, 2016). However, regarding many organizational practitioners’ main concern that whether pay dispersion among team members (i.e., horizontal pay dispersion within a team, HPD within a team) could help organizations achieve employee behavioral performance such as *team member work role performance* in the current uncertain, complex, and interdependent work systems, almost no effort and little attention has been directly dedicated to it. As an effort to bridge this research gap, we seek to examine why, when, and what effect HPD within a team tends to exert on *team member work role performance* from the perspective of the performance-shaping basis and team member’s workplace benign envy.

Due to the following reasons, we primarily focus on *performance-based HPD*, which was firstly put forward by Downes and Choi (2014). First, HPD has become more common in organizations. On the one hand, since teams have a potential synergy effect and excellent flexibility in the ongoing uncertain, complex, and interdependent context (Howard, 1995; Park et al., 2013), they have become many organizations’ substantial working unit and pay distribution unit (DeMatteo et al., 1998), which leads to more frequent horizontal pay comparison taking place among team members within the same hierarchy or performing similar jobs. On the other hand, organizational structure has become flat, which thereby means that many employees work at the same hierarchy and further exacerbates “horizontal” pay comparison. They combine to enable HPD, which refers to pay spread among team members within the same hierarchy or performing similar jobs (Shaw et al., 2002), to become more common. Second, employee attitudes toward HPD change over time. Nowadays in China, the new generation of employees, who were born after 1980, have already entered the workplace, have grown up under China’s higher education reform, have experienced China’s various economic changes, have enjoyed the benefits of China’s market economy, and have been influenced by both eastern and western cultures. Their upbringing may shape their attitudes (particularly the tolerance) toward HPD, which may be very different to their parents’. The new generation of employees may be more inclusive and more tolerant toward HPD, which may in turn affect the effects of HPD. Third, employee performance variation has become HPD’s most important shaping basis. To encourage employees to create high performance, many Chinese organizations have implemented a dispersed pay-for-performance plan. Employees have now universally accepted performance as one basis of organizational pay distribution, which has thereby made performance variation a legitimate source of HPD. Finally, it is the specific shaping basis that largely determines whether HPD is beneficial or detrimental (Gupta et al., 2012; Trevor et al., 2012). Consequently, we mainly focus on the performance-shaping basis of HPD, also known as performance-based HPD.

**FIGURE 1:**
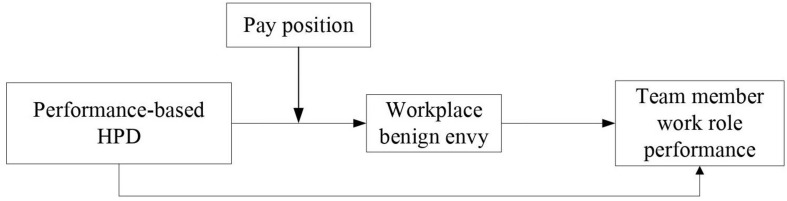
The theoretical model.

To sum up, why, when, and what effect performance-based HPD has on team member work role performance are of paramount importance in both practical and theoretical fields, which should receive greater attention. This study attempts to address them from the perspective of team member’s workplace benign envy. Specifically, according to emotion-related theory (Frijda, 1986; Roseman, 1996; Feather, 2006; Van de Ven et al., 2009, 2012), social comparison theory (Festinger, 1954), legitimacy theory (Aime et al., 2010), and expectation theory (Vroom, 1964), we propose that performance-based HPD could have an indirect-positive effect via workplace benign envy as well as a direct-positive effect on team member work role performance. Besides, we identify an important boundary condition (i.e., pay position) that is likely to strengthen the positive association between performance-based HPD and workplace benign envy and the indirect-positive effect of workplace benign envy on the basis of relative deprivation theory (Crosby, 1976). We posit that a team member’s higher pay position often signals his or her superiority in pay comparisons. This could enhance the legitimacy of performance-based HPD and the deservingness appraisal of others’ higher pay level, which in turn may elicit stronger workplace benign envy and ultimately may result in higher team member work role performance. The above arguments are summarized as a moderated mediation model in Figure 1, which were mainly supported by our multi-source survey data.

This study contributes to prior literature in three ways. Most notably, it concentrates on the practically and theoretically meaningful phenomenon in the current dynamic context—why, when, and what effect performance-based HPD may have on team member work role performance, which may plug the research gap that almost no study has dedicated attention to the effects of pay dispersion on employee behavioral performance. Second, whereas previous research (although it was scarce) mainly relied on team behavioral dynamics to explain pay dispersion effects, for example, the study of Ensley et al. (2007) mainly elaborated pay dispersion effects in TMTs (top management teams) through the mediating roles of cohesion, conflict, and group potency after which the mechanism research has almost made little progress, our research unravels the black box of pay dispersion effects from a new perspective of workplace benign envy. Thus, it not only enriches the outcome variables of pay dispersion but more importantly proposes a new mediating mechanism, which may help us better understand pay dispersion effects. Third, it identifies a boundary condition (i.e., pay position) that tends to affect the relationship between performance-based HPD and workplace benign envy and the mediating role of workplace benign envy.

The remainder of this paper is structured as follows. Section “Theoretical Background and Hypotheses” constructs a moderated mediation model and puts forward five hypotheses. Section “Materials and Methods” describes the sample, data procedure, measurement, and statistical analysis methods in details. Section “Results” presents the hypotheses tests results and findings. Section “Discussion” discusses theoretical and practical implications, limitations, and suggestions for future research.

## Theoretical Background and Hypotheses

Horizontal pay dispersion (i.e., HPD) within a team refers to pay disparity (pay dispersion, pay gap, pay spread, pay differential, or pay variation) among team members within the same hierarchy or performing similar jobs, which is mainly the result of team members’ differences in performance, abilities, skills, knowledge, seniority, and political behavior, discrepancies of supervisors’ appraisal preference, random decision in pay distribution, computing error, and other unknown factors (Gupta et al., 2012). In the context of Chinese organizations, pay for key human capital inputs and pay for performance are so widespread that differences in performance and some key human capital inputs (such as gender, age, seniority, education, professional or skill title, and marital status) are well-recognized sources of HPD by both employees and employers. Therefore, the performance-shaping basis and the human capital input-shaping basis are both legitimate (Aime et al., 2010), according to which we could subdivide HPD into three separate parts: *performance-based HPD*, *human capital input-based HPD*, and *other(s)-based HPD.*

*Performance-based HPD* involves the pay differential primarily resulting from performance differences in workers who are in the same hierarchy or are performing similar jobs. *Human capital input-based HPD* differentiates the part which is mainly the result of team members’ differences in some key human capital inputs from the overall HPD. *Other(s)-based HPD* emphasizes the pay spread which mainly arises from distinctions in team members’ political behaviors, differences of supervisors’ performance appraisal preference, random decisions in pay distribution, computing errors, or other unknown factors. Here, we are committed to exploring the potential effect of *performance-based HPD* on team member work role performance, and will control for the influences of the other two.

### Performance-Based HPD, Workplace Benign Envy, and Team Member Work Role Performance

In view of the functional approach to emotions (Frijda, 1986) and emotion appraisal theory (Roseman, 1996), workplace envy, which is defined as a frustrating and painful emotion that an individual is more likely to experience when he or she observe that his or her coworkers possess something important to self-concept whereas the individual does not (Parrott and Smith, 1993; Cohen-Charash and Mueller, 2007), can be classified into two types: workplace benign envy and workplace malicious envy, which are distinct in their emotional elements, attention bias, and action tendencies (Van de Ven et al., 2009; Tai et al., 2012; Van de Ven, 2016). Specifically, workplace benign envy often relates to affection and admiration, which in turn could motivate an individual toward opportunities to improve/enhance himself or herself, whereas, workplace malicious envy is usually associated with suspicion and hostility, which in turn could encourage an individual to focus on the coworker he or she envies and ultimately may result in a pulling-down motivation to damage that coworker (Van de Ven et al., 2009; Van de Ven, 2016). Here, we primarily focus on workplace benign envy and seek to reveal the relationships between performance-based HPD, workplace benign envy, and team member work role performance based on emotion-related theory (Frijda, 1986; Roseman, 1996; Feather, 2006; Van de Ven et al., 2009, 2012), social comparison theory (Festinger, 1954), legitimacy theory (Aime et al., 2010), expectation theory (Vroom, 1964), and the extant research (Parrott and Smith, 1993; Cohen-Charash and Mueller, 2007; Kepes et al., 2009; Van de Ven et al., 2009, 2012; Shaw, 2014).

By the definition of workplace envy (Parrott and Smith, 1993; Cohen-Charash and Mueller, 2007) and the insights of social comparison theory (Festinger, 1954), we predict that performance-based HPD within a team may be one of the possible antecedents that could activate a team member’s workplace envy, whether it is in the benign or malicious form. Specifically, an individual is more inclined to compare himself or herself with someone close to or similar to him or her in the domains of self-concept (Salovey and Rodin, 1984) or self-evaluation (Festinger, 1954), and, if it is an unfavorable social comparison that directly threatens his or her professional identity, the individual is more likely to experience a frustrating and painful emotion that may engender a moving-up or pull-down motivation to reduce his or her pain, which eventually may activate his or her workplace envy (Parrott and Smith, 1993; Cohen-Charash and Mueller, 2007). In terms of performance-based HPD within a team, it is exactly the type of comparison among similar team members at the performance-based pay level which is well established as an important domain associated with self-concept (Hill et al., 2011). Besides, the self-evaluation need of an individual will unconsciously motivate him or her to frequently compare his or her performance-based pay level with that of a coworker who is close to or similar to him or her (Festinger, 1954). Hence, performance-based HPD is more likely to elicit a team member’s workplace envy.

As aforementioned, we attempt to explore the possible effect of performance-based HPD on workplace benign envy rather than on the broad kind of workplace envy. Next, we further examine the specific relationship between performance-based HPD and workplace benign envy. According to the extant literature (Van de Ven et al., 2009, 2012), we propose that a team member’s *(un)deservingness appraisal* of others’ higher pay level and *control potential perception* to improve his or her own relatively lower pay level combine to determine whether his or her workplace benign envy will occur in the situation of large performance-based HPD. Specifically, a team member’s *(un)derservingness appraisal* refers to the extent to which he or she consider the higher pay level of others as deservingness or underservingness (Feather, 2006). A team member’s *control potential perception* depicts the degree to which a team member believes he or she could do something to improve his or her current lower pay level (Van de Ven et al., 2012). Large performance-based HPD within a team tends to drive a team member who is disadvantaged in a pay comparison to activate his or her appraisal processes of (un)derservingness and control potential simultaneously. If the (un)derservingness appraisal of others’ higher pay level tends to be deservingness and the individual is more inclined to believe that he or she has a higher control potential to make the current unfavorable pay comparison better, then, in this situation, large performance-based HPD within a team is more likely to give rise to workplace benign envy. Otherwise, either the underservingness appraisal of others’ higher pay level or a lower control potential perception is more likely to result in workplace malicious envy. Furthermore, it is confirmed that an individual’s appraisal of others’ higher pay level is closely related to the subjective (un)fairness of pay distribution (Smith et al., 1994), which largely depends on the legitimacy of the specific pay shaping basis (Aime et al., 2010). Since performance-based HPD within a team is widely and well recognized (Kepes et al., 2009; Shaw, 2014), it is of high legitimacy (Aime et al., 2010), which in turn may enable an individual team member to find the higher pay level of others, based on performance, deserving. Besides, the close performance-pay linkage within performance-based HPD has provided the individual team member with a feasible way to improve his or her current unfavorable pay level, which therefore may bestow a higher control potential perception. Taking the above together, we infer that performance-based HPD within a team is more likely to promote workplace benign envy, and, the larger the performance-based HPD within a team is, the stronger the workplace benign envy may be. Hence, we put forward Hypothesis 1.

*Hypothesis 1*: Performance-based HPD within a team tends to be positively related to a team member’s workplace benign envy.

As we discussed earlier, workplace benign envy is a kind of painful emotion. It has been confirmed by studies in neuroscience that workplace envy (benign and malicious) actually activates pain neuronal regions in the brain (Takahashi et al., 2009). Moreover, prior literature has found that, when an individual is experiencing benign envy, it often implies that he or she has already prepared some challenge-oriented actions to reduce the pain (Craig, 2003; MacDonald and Leary, 2005). Compared to a team member with stronger workplace malicious envy, a team member with stronger workplace benign envy is more likely to observe an opportunity of self-improvement from the unfavorable comparison and is more inclined to employ some challenge-oriented actions that concentrate on the comparative advantage of the coworker and aim at self-improving (Van de Ven et al., 2009). As discussed earlier, in the current complex and dynamic context where teams are organizations’ substantial working unit, employee performance not only depends on to what extent an employee has completed the tasks and job responsibilities clearly prescribed in the static job description but is also contingent on whether he or she and other members could effectively cooperate with each other (i.e., team member work role behaviors with proficiency), quickly adapt to various changes inside and outside (i.e., team member work role behaviors with adaptivity), and proactively make some innovative suggestions for teamwork (i.e., team member work role behaviors with proactivity) (Griffin et al., 2007). Thus, the challenge-oriented actions of a team member with stronger workplace benign envy will transform into actions strengthening his or her team member work role behaviors with proficiency, adaptivity, and proactivity in the situation of large performance-based HPD. Such that the stronger the workplace benign envy incurred by larger performance-based HPD is, the greater the challenge-oriented action tendency is, and the stronger the motivation to perform team member work role behaviors with proficiency, adaptivity, and proactivity becomes, which ultimately may boost team member work role performance. Accordingly, we posit that workplace benign envy tends to play a mediating role in the relationship between performance-based HPD and team member work role performance. Thus, we raise Hypothesis 2.

*Hypothesis 2*: Performance-based HPD indirectly promotes team member work role performance by activating a team member’s workplace benign envy.

Regarding the relationships between performance-based HPD and team member work role performance, we have discussed the possible indirect-positive relationship between them via workplace benign envy so far. Next, we attempt to infer the potential direct-positive effect of performance-based HPD on team member work role performance according to expectation theory. Expectation theory stipulates that the high valence of outcome, the close linkage between effort and performance, and the highly related association between performance and outcome combine to determine an employee’s performance motivation (Vroom, 1964). As for our study, here, a higher pay level is the outcome. Each team member expects a higher pay level [whereas his or her employer expects higher (outcome) performance], which signifies that the first condition (i.e., the high valence of outcome) is met. The pay-for-performance system within a team is an effective tool that organizations can adopt to reach both of these expectations as well as to satisfy the other two conditions through the close (outcome) performance-pay linkage and the highly related association between effort and (outcome) performance. Therefore, according to expectation theory, performance-based HPD may strengthen behavioral motivation toward higher (outcome) performance. As employee (outcome) performance not only relies on individual efforts but also depends on team-oriented work role behaviors with proficiency, adaptivity, and proactivity in the uncertain, complex, and interdependent work systems (Griffin et al., 2007), the individual behavioral motivation toward higher (outcome) performance will in turn develop into motivation toward team-oriented work role behaviors with proficiency, adaptivity, and proactivity as a team member. And, the larger the performance-based HPD within a team is, the stronger the behavioral motivation toward higher (outcome) performance will become, and the higher probability of (outcome) performance motivation developing into motivation toward team-oriented work role behaviors with proficiency, adaptivity, and proactivity, which ultimately may enhance team member work role performance. Accordingly, performance-based HPD within a team is likely to be positively related to team member work role performance. Hence, we put forward Hypothesis 3.

*Hypothesis 3*: Performance-based HPD within a team is directly and positively related to team member work role performance.

### Moderating Role of Pay Position

It is established that workplace benign envy is a function of many contextual factors, such as a broad kind of social comparison and the specific comparison characteristics (Salovey and Rodin, 1984; Smith et al., 1999; Lange and Crusius, 2015), competitive organizational climate (Smith, 2000; Vecchio, 2005), and leader-member exchange relationship (Vecchio, 2005; Kim et al., 2010), which enables us to predict that the activating effect of performance-based HPD on workplace benign envy and the mediating role of workplace benign envy may vary according to the specific context. Here, we focus on the possible moderating role of pay position, as it usually represents one important pay comparison characteristic and is confirmed to be key to how an individual employee reacts toward pay dispersion (Pfeffer and Langton, 1993; Trevor and Wazeter, 2006; Gupta et al., 2012).

A team member’s pay position refers to his or her pay level relative to other members within a team. A higher pay position often implies a pay comparison advantage over others, which may promote a feeling of fairness in pay. Whereas a lower pay position usually represents a disadvantage in pay comparison, which may lead to a deprivation feeling and the perception of unfairness in pay (Trevor and Wazeter, 2006). Relative deprivation theory argues that the deprivation and pay unfairness feeling will be greater when pay dispersion is large (Crosby, 1976), based on which we posit that pay position may moderate the activating effect of performance-based HPD on workplace benign envy and the mediating role of workplace benign envy. Specifically, when a team member is in a higher position in the team pay distribution, large performance-based HPD within the team tends to strengthen the perception of a pay comparison advantage over others, which thereby may bring pay fairness and encourage the individual to be more inclined to view a higher pay level in the situation of large performance-based HPD as deservingness (Trevor and Wazeter, 2006). In addition, the close linkage between performance and pay, to some extent, presents a feasible way to enhance pay level and to retain the current pay comparison advantage, which thereby could heighten the control potential perception. Thus, for a team member who is in a higher pay position, the deservingness of a higher pay level in a large performance-based HPD situation and the heightened control potential perception combine to give rise to a much stronger activating effect of performance-based HPD on workplace benign envy. And, for the sake of retaining the current pay comparison advantage, a team member within stronger workplace benign envy should therefore be more strongly motivated to focus on team-oriented work role behaviors with proficiency, adaptivity, and proactivity which will create the expected level of performance and thereby will bring a higher pay level in the ongoing uncertain, complex, and interdependent work systems within a large performance-based HPD scenario. By contrast, when a team member is in a lower position in the team pay distribution, large performance-based HPD within a team may result in a disadvantageous perception in pay comparison, exacerbate the deprivation feeling in pay distribution, and engender strong pay unfairness (Trevor and Wazeter, 2006; Gupta et al., 2012), which thereby may lower the deservingness appraisal of a higher pay level in a large performance-based HPD situation and weaken workplace benign envy. It is noteworthy that since the well-recognized legitimacy of the performance-shaping basis and its decisive role in (un)deservingness appraisal, here it is lower and weaker more than others. Thus, for a team member who is in a lower pay position, the positive activating effect of performance-based HPD on workplace benign envy is likely to be weaker, and, a team member with weaker workplace benign envy should be less motivated to improve his or her behavioral and outcome performance due to a relatively lower level of deservingness appraisal. Taking the above together, when a team member is in a higher rather than lower pay position, large performance-based HPD within a team is more inclined to strengthen workplace benign envy and thereby may bring higher team member work role performance. Therefore, we propose Hypotheses 4 and 5.

*Hypothesis 4:* A team member’s pay position moderates the positive association between performance-based HPD and his or her workplace benign envy such that the positive association is much stronger when a team member is in a higher rather than lower pay position.
*Hypothesis 5:* A team member’s pay position moderates the indirect-positive effect of performance-based HPD on team member work role performance via workplace benign envy such that the indirect-positive effect is much stronger when a team member is in a higher rather than lower pay position.

The moderated mediation model is summarized in Figure 1.

## Materials and Methods

### Sample and Procedure

The extant literature suggests two significant trends with respect to empirical research samples and data collecting. First, focus has gradually shifted from sports teams to top management teams in large-scale listed companies, and recently to ordinary employee teams in non-listed companies, which enables empirical research samples to be more diversified and of greater representativeness. Second, many researchers have begun to adopt a combined method to collect data, such that they tend to obtain objective data (i.e., pay level and outcome performance) from employers and subjective data in terms of employees’ psychological, emotional, or behavioral reactions from employees or direct supervisors using scale-evaluation, which not only goes beyond early researchers’ over-reliance on the market-open data but more importantly promotes the influencing mechanism research of pay dispersion effects because of the subjective data collecting.

Following the above two trends, we pay close attention to teams mainly constituted by ordinary employees in non-listed companies and employ the combined data-collecting method. Since it is difficult to collect ordinary employees’ individual-level objective pay data, which is often kept confidential by organizations, we conducted surveys in 16 organizations that had previously cooperated with us. The 16 organizations are located in Jiangsu Province of China and are scattered across a wide range of industries, including sales, manufacturing, government, education, logistics, finance, research and development, power, medical services, and insurance. In each organization, two to six ordinary employee teams were randomly selected. Then, team leaders or human resource managers were requested to provide objective data about each team member’s pay level (*year t−1*) and performance (*year t−2*); team leaders were also asked to rate each subordinate’s team member work role performance (*year t*) using scale-evaluation. Meanwhile, each team member was asked to provide information about his or her own human capital inputs (*year t−2*) (i.e., gender, age, education, seniority, professional or skill title, and marital status) and self-rate his or her own workplace benign envy (*year t*) with scale-evaluation. It is noteworthy that we strictly stipulated the year requirements of objective data [i.e., each team member’s pay level (*year t−1*), performance (*year t−2*), and human capital inputs (*year t−2*)], which were used to estimate the three subdivided parts/types of pay level (*year t−1*) to ensure a clear causal relationship between the predicted variables [i.e., the three subdivided parts/types of pay level (*year t−1*)] and the predictors [i.e., each team member’s performance (*year t−2*) and human capital inputs (*year t−2*)].

All the surveys were finished in 6 months. We gathered the scale-evaluation questionnaires separately from team leaders and team members and matched them with objective data [i.e., team member’s pay level (*year t−1*), performance (*year t−2*), and human capital inputs (*year t−2*)] by the assistance of team leaders or human resource managers. A total of 511 team members within 82 teams took part in our surveys. A total of 149 participants within 16 teams were deleted due to their unmatched or incomplete data. Ultimately, we obtained 362 valid participants within 66 teams. As shown in Table 1, 21.212, 10.606, 21.212, 22.727, and 24.242% of the 66 teams, respectively, came from sales, manufacturing, government, education, and others (such as logistics, finance, research and development, power, medical services, and insurance). Team scales ranged from 3 to 16 members (excluding team leaders), with an average of six members (mean = 5.485). The participants (*N* = 362) were 51.105% male and 48.895% female, with 72.099% married; 63.812% were under 35 years old; 73.481% had worked for no more than 10 years; 92.818% had finished vocational college or university; and 88.398% were below a senior-level in profession or skill title.

**TABLE 1:** Sample details.

Team-level (*N* = 66)							
Variable	Value	*N*	Ratio (%)	Variable	Value	*N*	Ratio (%)
Industry	Sales	14	21.212	Industry	Power	3	4.545
	Manufacturing	7	10.606		Medical services	3	4.545
	Government	14	21.212		Insurance	2	3.030
	Education	15	22.727	Team scale	<5	15	22.727
	Logistics	3	4.545		5	23	34.848
	Finance	2	3.030		6–8	26	39.394
	Research and development	3	4.545		>9	9	3.030

**Team member-level (*N* = 362)**							

Gender	Male	185	51.105	Marital status	Married	261	72.099
	Female	177	48.895		Single	101	27.901
Education	High school or below	26	7.182	Seniority	<2 years	50	13.812
	Vocation college	115	31.768		2–5 years	141	38.950
	Undergraduate in university	116	32.044		6–10 years	75	20.718
	Graduate in university	105	29.006		11–20 years	59	16.298
Age	<25 years	57	15.746		>20 years	37	10.221
	25–30 years	109	30.110	Professional or skill title	Assistant	143	39.503
	31–35 years	65	17.956		Intermediate	177	48.895
	36–50 years	109	30.110		Associate senior	38	10.497
	>50 years	22	6.077		Full senior	4	1.105

### Measurement

#### Independent Variable

To estimate the independent variable: performance-based HPD (*year t−1*) and two control variables: human capital input-based HPD (*year t−1*) and other(s)-based HPD (*year t−1*), we at first decomposed each team member’s pay level (*year t−1*) into performance-based pay level (*year t−1*), human capital input-based pay level (*year t−1*), and other(s)-based pay level (*year t−1*) adopting the method of Trevor et al. (2012) and then used the difference measuring approach of Henderson and Fredrickson (2001). Specifically, we predicted three separate parts of each team member’s pay level (*year t−1*) according to individual-level regression equations of performance (*year t−2*) and human capital inputs (*year t−2*) separately and together on pay level (*year t−1*) (show in Table 2) and then obtained each team member’s performance-based HPD *(year t−1)*, human capital input-based HPD (*year t−1*), and other(s)-based HPD (*year t−1*) through calculating the difference values separately between his or her performance-based pay level *(year t−1)* and the team’s average performance-based pay level *(year t−1)*, his or her human capital input-based pay level *(year t−1)* and the team’s average human capital input-based pay level *(year t−1)*, and his or her other(s)-based pay level *(year t−1)* and the team’s average other(s)-based pay level *(year t−1)*.

**TABLE 2:** Individual-level regression equations of performance in *year t−2* (*X*_1_) and human capital inputs in *year t−2* (*X*_2_–*X*_7_) separately and together on pay level in *year t−1* (*Y*) (*N* = 362).

Independent variable	Regression equation	*R*	*R*^2^	*F*
*X*_1_: performance (*year t−2)*	*Y* = 9.206 + 0.218*X_1_	0.616	0.380	195.884***
*X*_2_–*X*_7_: human capital inputs (*year t−2*)	*Y* = 10.582 + 0.132*X_2_− 0.007*X_3_ + 0.058*X_4_ + 0.000*X_5_ + 0.362*X_6_− 0.053*X_7_	0.598	0.357	32.896***
*X*_1_–*X*_7_: performance (*year t−2*) and human capital inputs (*year t−2*)	*Y* = 8.920 + 0.191*X_1_ + 0.071*X_2_−0.004*X_3_ + 0.089*X_4_ + 0.006*X_5_ + 0.201*X_6_−0.046*X_7_	0.783	0.613	70.904***

***p < 0.01. *X*_1_, *X*_2_, *X*_3_, *X*_4_, *X*_5_, *X*_6_, *X*_7_, and *Y*, respectively, represent team member’s performance, gender, age, education, seniority, professional or skill title, marital status in *year t−2*, and pay level in *year t−1*. Gender (*X*_2_): 0 = female, 1 = male; education (*X*_4_): 1 = high school or below, 2 = vocational college, 3 = undergraduate in university, 4 = graduate in university; professional or skill title (*X*_6_): 1 = assistant, 2 = intermediate, 3 = associate senior, 4 = full senior; marital status (*X*_7_): 0 = single, 1 = married.

#### Dependent Variable

Team member work role performance (*year t)* is the dependent variable. We used a nine-item scale (see Appendix) developed by Griffin et al. (2007) to measure it. To avoid the issue of common method variance (CMV), we asked team leaders to rate each subordinate’s team member work role performance using a five-point Likert rating method (1 = strongly disagree, 5 = strongly agree). The Cronbach alpha for this scale is 0.888.

#### Mediator

Workplace benign envy (*year t*) is the hypothesized mediator. We adapted the benign envy scale of Lange and Crusius (2015) to the context of Chinese organizations’ pay distribution. Each team member was asked to indicate to what extent the five items (see Appendix) described how he or she experienced or felt in the workplace with a five-point Likert rating method (1 = not at all, 5 = extremely). The Cronbach alpha for this scale is 0.832.

#### Moderator

Team member’s pay position (*year t−1*) is the hypothesized moderator. According to Pfeffer and Davis-Blake (1992), we calculated the ratio of each team member’s pay level (*year t−1*) to team’s average pay level (*year t−1*) to measure it.

#### Control Variables

As different sources of HPD could result in different employee psychology, behaviors, and performance (Trevor and Wazeter, 2006; Kepes et al., 2009), we controlled for human capital input-based HPD (*year t−1*) and other(s)-based HPD (*year t−1*) in data analysis. Regarding some demographic variables which are confirmed to be related to workplace envy (Lange and Crusius, 2015) and team member work role performance (Griffin et al., 2007) and should be controlled for, since some variables representing team member’s human capital inputs (*year t−2*) such as gender, age, seniority, education, professional or skill title, and marital status were already used to predict his or her human capital input-based pay level (*year t−1*) and to calculate human capital input-based HPD (*year t−1*), we chose to control for human capital input-based HPD (*year t−1*) rather than the demographic variables that had already been included by the former one. Moreover, our participants came from a variety of industries such as sales, manufacturing, government, education, and others. Since employees in different industries may demonstrate different levels of workplace benign envy and team member work role performance, we created four industry dummies (industry 1, industry 2, industry 3, and industry 4) and controlled for them as well in data analysis.

### Statistical Analysis

Other than relying on hierarchical regression analysis within SPSS to test the mediation of workplace benign envy according to the procedures recommended by Baron and Kenny (1986), we used the Sobel test and a Bootstrapping approach in PROCESS to confirm whether the moderated mediation was established (Hayes and Preacher, 2010). We at first did reliability and validity analysis of the two latent variables in the theoretical model (i.e., workplace benign envy and team member work role performance in Figure 1) using MPLUS.

## Results

### Reliability and Validity Analysis

At first, we did confirmatory factor analysis (CFA) using MPLUS to confirm the discriminant validity of the two latent variables. The CFA results show that the hypothesized two-factor model has a good fit for the data (Hu and Bentler, 1999): χ^2^(8) = 19.331, χ^2^/df = 2.416, CFI = 0.990, TLI = 0.981, RMSEA = 0.063, SRMR = 0.027. Besides, it is much better than the one-factor model (i.e., workplace benign envy + team member work role performance model) as the Chi-square difference is significant at the 0.001 level. Based on the standardized loading values of the two-factor model, we further calculated the composite reliability (CR), the average variance extraction (AVE), and the square root of AVE of each variable, results of which are presented in Table 3. As shown, the Cronbach’s alpha (see in section “Measures”) and CR of the two latent variables exceed 0.70 (O’Leary-Kelly and Vokurka, 1998); the AVE values exceed 0.50 (Fornell and Larcker, 1981); and none of the correlation coefficients separately between workplace benign envy/team member work role performance and the other nine variables exceed the corresponding square root of AVE (Hair et al., 2010). Therefore, the two latent variables are confirmed to have good reliability and validity.

**TABLE 3:** Means, standard deviations, and Pearson correlations among variables (*N* = 362).

Variables	1	2	3	4	5	6	7	8	9	10
1. Human capital input-based HPD	–									
2. Other(s)-based HPD	0.597**	–								
3. Industry 1	*−*0.126*	*−*0.160**	–							
4. Industry 2	*−*0.014	0.072	*−*0.176**	–						
5. Industry 3	0.403**	0.349**	*−*0.279**	*−*0.176**	–					
6. Industry 4	*−*0.057	0.022	*−*0.293**	*−*0.184**	*−*0.293**	–				
7. Performance-based HPD	0.260**	0.240**	0.091	0.068	0.074	*−*0.258**	–			
8. Pay position	0.593**	0.855**	*−*0.032	0.060	0.249**	*−*0.095	0.572**	–		
9. Workplace benign envy	*−*0.169**	*−*0.194**	0.185**	0.009	*−*0.209**	0.078	0.052	0.104*	(0.841/0.640/0.800)	
10. Team member work role performance	0.024	0.003	0.168**	0.009	*−*0.054	0.036	0.100	0.072	0.426**	(0.879/0.708/0.841)
Mean	*−*0.033	*−*0.094	0.218	0.099	0.218	0.235	*−*1.208	1.000	3.972	4.075
SD	0.264	0.447	0.414	0.300	0.414	0.424	1.983	0.041	0.615	0.456

**p < 0.01, *p < 0.05. The three estimate values on the diagonal in parentheses are, respectively, CR, AVE, and the square root of AVE. Industry, 1 = sales, 2 = manufacturing, 3 = government, 4 = education.

### Correlation Analysis

Besides CR, AVE, and the square root of AVE, Table 3 summarizes the means, standard deviations, and Pearson correlations among variables. As shown, team member work role performance is not significantly associated with performance-based HPD (*r* = 0.100, *p* > 0.05, n. s.) whereas it is positively related to workplace benign envy (*r* = 0.426, *p* < 0.01). Workplace benign envy is not significantly related to performance-based HPD (*r* = 0.052, *p* > 0.05, n. s.) whereas it is significantly correlated with human capital input-based HPD (*r* = *−*0.169, *p* < 0.01), other(s)-based HPD (*r* = *−*0.194, *p* < 0.01), and pay position (*r* = 0.104, *p* < 0.05).

### Hypotheses Tests

We mainly ran hierarchical regression analysis to test Hypotheses 1, 2, 3, and 4. To further test the moderated mediation of workplace benign envy (i.e., Hypotheses 2 and 5), we used PROCESS to run the Sobel test and the Bootstrapping estimation. Table 4 presents the results.

**TABLE 4:** Results of hierarchical regression analysis (*N* = 362), Sobel test, and Bootstrapping estimation.

Variable	Mediator: workplace benign envy (*M*_*E*_)				Dependent variable: team member work role performance (*Y*)			
	Model 1	Model 2		Model 3	Model 4	Model 5	Model 6	Model 7
Intercept	3.832 (0.069)***	3.856 (0.070)***		4.102 (2.485)	3.951 (0.053)***	3.966 (0.045)***	2.749 (0.149)***	2.778 (0.151)***
**Controls**								
Human capital input-based HPD	*−*0.094 (0.152)	*−*0.153 (0.153)		*−*0.161 (0.155)	0.089 (0.116)	0.050 (0.117)	0.118 (0.106)	0.097 (0.107)
Other(s)-based HPD	*−*0.185 (0.090)*	*−*0.219 (0.090)*		*−*0.213 (0.189)	*−*0.019 (0.068)	*−*0.041 (0.069)	0.039 (0.063)	0.026 (0.064)
Industry 1	0.304 (0.093)**	0.302 (0.093)**		0.286 (0.092)**	0.273 (0.071)***	0.271 (0.070)***	0.177 (0.066)	0.178 (0.065)**
Industry 2	0.153 (0.120)	0.160 (0.120)		0.156 (0.120)	0.139 (0.091)	0.144 (0.091)	0.091 (0.084)	0.094 (0.084)
Industry 3	*−*0.050 (0.103)	*−*0.019 (0.103)		0.001 (0.104)	0.066 (0.078)	0.086 (0.079)	0.082 (0.071)	0.092 (0.072)
Industry 4	0.207 (0.093)*	0.264 (0.095)**		0.255 (0.095)**	0.157 (0.071)*	0.194 (0.073)**	0.093 (0.065)	0.113 (0.067)
**Independent variable**								
Performance-based HPD		0.041 (0.017)*		0.052 (0.026)*		0.026 (0.013)*		0.014 (0.012)
**Moderator**								
Pay position				0.276 (2.445)				
**Interaction**								
Performance-based HPD × pay position				0.080(0.033)*				
**Mediator**								
Workplace benign envy							0.314 (0.037)***	0.308 (0.037)***
F	5.827***	5.877***		5.417***	2.826*	3.032**	13.311***	11.824***
Adjust R^2^	0.074***	0.086*		0.099*	0.029*	0.038*	0.193***	0.193***

**Sobel test and Bootstrapping estimation results of the mediation of workplace benign envy (Hypothesis 2)**								

			**Value**		**z**		**p**	

Sobel test			0.013 (0.006)		2.282		0.023	

**Bootstrapping estimation**			**Effect**		**LL 95% CI**		**UL 95% CI**	

Indirect effect of workplace benign envy			0.013 (0.006)		0.002		0.025	
Direct effect			0.014 (0.012)		*−*0.010		0.037	

**Bootstrapping estimation results of the moderated mediation across different levels of pay position (Hypothesis 5)**								

**Levels**			**Conditional indirect effect**		**LL 95% CI**		**UL 95% CI**	

Lower pay position (0.959)			0.004 (0.008)		*−*0.011		0.019	
Higher pay position (1.041)			0.029 (0.013)		0.004		0.056	

*p < 0.05, **p < 0.01, ***p < 0.001. The statistics reported are the unstandardized regression coefficients with standard errors in parentheses. Industry, 1 = sales, 2 = manufacturing, 3 = government, 4 = education. CI, confidence interval; LL, lower limit; UL, upper limit. Bootstrapping sample size = 5,000.

After controlling for human capital input-based HPD, other(s)-based HPD, and industry dummies in Model 1, the results of Model 2 suggest that performance-based HPD is positively related to workplace benign envy (β = 0.041, *p* < 0.05), which thus supports Hypothesis 1.

Regarding Hypothesis 2, as shown in Models 2, 5, 6, and 7, (1) performance-based HPD (i.e., the independent variable *X*) is positively associated with workplace benign envy (i.e., the mediator *M*_*E*_) (β = 0.041, *p* < 0.05 in Model 2) and team member work role performance (i.e., the dependent variable *Y*) (β = 0.026, *p* < 0.05 in Model 5); (2) workplace benign envy (*M*_*E*_) is positively related to team member work role performance (*Y*) (β = 0.314, *p* < 0.001 in Model 6); and (3) after the hypothesized mediator (i.e., workplace benign envy) entered the regression model, the regression coefficient of performance-based HPD (*X*) on team member work role performance (*Y*) becomes insignificant (β = 0.026, *p* < 0.05 in Model 5→β = 0.014, *p* > 0.05, n.s. in Model 7). Thus, following the three-step testing procedures (Baron and Kenny, 1986), the mediating role of workplace benign envy predicted by Hypothesis 2 is supported. We further conducted the Sobel test and the Bootstrapping estimation using PROCESS (Hayes and Preacher, 2010). As shown in Table 4, the indirect effect of performance-based HPD (*X*) on team member work role performance (*Y*) via workplace benign envy (*M*_*E*_) is significantly positive (the indirect effect = 0.013, Sobel *z* = 2.282, *p* = 0.023; 95% CI [0.002, 0.025], excluding 0). Hence, Hypothesis 2 is established.

As shown in Model 5, performance-based HPD (*X*) is positively related to team member work role performance (*Y*) (β = 0.026, *p* < 0.05), which seemingly supports Hypothesis 3. However, it is exactly the overall effect of performance-based HPD on team member work role performance rather than the direct effect that Hypothesis 3 predicts. After controlling for workplace benign envy (*M*_*E*_), the regression coefficient of performance-based HPD (*X*) on team member work role performance (*Y*) in Model 7 exactly represents the direct effect, which suggests that performance-based HPD (*X*) is not significantly related to team member work role performance (*Y*) (β = 0.014, *p* > 0.05, n. s.). The results of the Bootstrapping estimation do not support Hypothesis 3 either (the direct effect = 0.014, 95% CI [*−*0.010, 0.037], including 0). Thus, Hypothesis 3 is not established.

**FIGURE 2:**
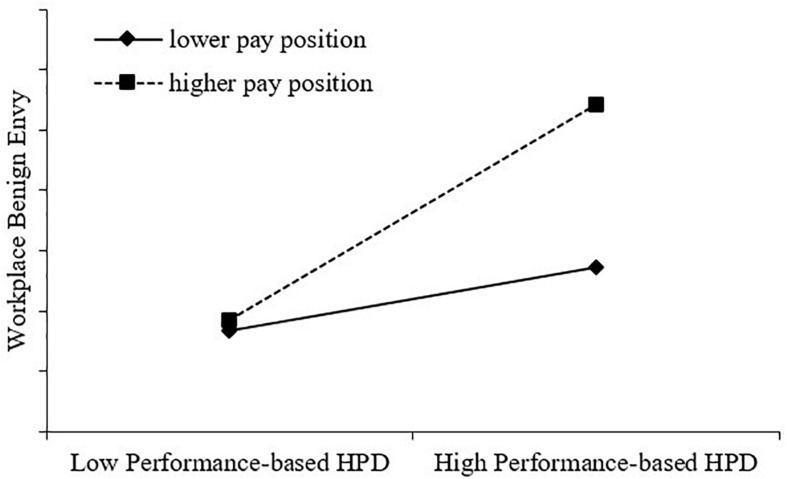
Moderation by pay position.

Models 2 and 3 show the testing result of Hypothesis 4, such that performance-based HPD (*X*) is indeed positively associated with workplace benign envy (*M*_*E*_) (β = 0.041, *p* < 0.05 in Model 2) and performance-based HPD (*X*) and pay position (i.e., the hypothesized moderator M_*O*_) interact with each other (i.e., performance-based HPD × pay position) to significantly predict workplace benign envy (*M*_*E*_) (β = 0.080, *p* < 0.05 in Model 3), which initially supports Hypothesis 4. According to the extant literature (Zhang and Sun, 2020), we did a simple slope test of the association between performance-based HPD (*X*) and workplace benign envy (*M*_*E*_) separately at a higher pay position (1 SD above the mean) and a lower pay position (1 SD below the mean). As shown in Figure 2, the positive association between performance-based HPD (*X*) and workplace benign envy (*M*_*E*_) is much stronger when pay position (M_*O*_) is higher (β = 0.452, *t* = 2.555, *p* = 0.011) rather than lower (β = 0.132, *t* = 2.620, *p* = 0.009). Thus, Hypothesis 4 is established.

Hypothesis 5 predicts that team member’s pay position also moderates the mediation of workplace benign envy such that the indirect-positive effect of performance-based HPD on team member work role performance via workplace benign envy is much stronger when team member’s pay position is higher rather than lower. As shown in Table 4, the Bootstrapping estimation results of the moderated mediation across different levels of pay position indicate that when team member’s pay position is higher (1 SD above the mean, i.e., 1.041), the indirect effect of performance-based HPD on team member work role performance via workplace benign envy is significantly positive (the conditional indirect effect = 0.029, 95% CI [0.004, 0.056], excluding 0), whereas, when team member’s pay position is lower (1 SD below the mean, i.e., 0.959), the indirect effect is not significant (the conditional indirect effect = 0.004, 95% CI [*−*0.011, 0.019], including 0). Accordingly, Hypothesis 5 is established.

## Discussion

As work systems have become increasingly uncertain, complex, and interdependent, two major changes are taking place silently. First, many organizations are adopting teams as their substantial working unit to enhance the flexibility in such a complex and dynamic context, which has challenged the traditional view of employee performance and has made team member work role performance, which could fully account for employee team-oriented behaviors with proficiency, adaptivity, and proactivity as a team member, become the tenet of employee performance. Second, due to the prevalence of teams and the significance of team member work role performance, many organizations expect to achieve team member work role performance through implementing a dispersed pay-for-performance plan within a team, which thereby leads to the common existence of performance-based HPD. The two major changes combine to give rise to organizational practitioners’ main concern of whether performance-based HPD could actually affect team member work role performance. Unfortunately, almost no effort and little attention has been dedicated to it. This study focuses on this concern and further explores why and when performance-based HPD within a team could play a positive role in achieving team member work role performance in the perspective of employees’ emotional reactions toward pay dispersion (i.e., workplace benign envy), which are crucial yet underexplored due to the great difficulty in collecting both objective and subjective data. The findings suggest that organizational benefits from large performance-based HPD within a team may be even more than previously revealed (Han et al., 2015), as organizations could achieve team member work role performance through the positive mediating role of workplace benign envy in a large performance-based HPD situation. Moreover, the positive association between performance-based HPD and workplace benign envy and the mediating role of workplace benign envy are much stronger when a team member’s pay position is higher rather than lower.

However, Hypothesis 3, which predicts the direct-positive effect of performance-based HPD on team member work role performance, is not established. We argue that two important factors may lead to it. First, we put forward Hypothesis 3 on the basis of expectation theory (Vroom, 1964) and employees’ shared cognition that employee (outcome) performance largely depends on employee team-oriented behaviors with proficiency, adaptivity, and proactivity as a team member in such a dynamic context with the prevalence of teams (Griffin et al., 2007). Put differently, only when each employee is fully aware of the critical role of team member work role behaviors in achieving his or her (outcome) performance, could his or her motivation toward (outcome) performance generated by large performance-based HPD according to expectation theory develop into motivation toward team member work role behaviors, which thereby may bring a direct-positive effect to performance-based HPD on team member work role performance. Otherwise, either a lack of or insufficient group cognition may fail to give rise to the direct-positive effect, because higher employee (outcome) performance not only relies on individual employee’s team member work role behaviors but also depends on his or her coworkers’ team member work role behaviors (Griffin et al., 2007). Second, by definition, team member work role performance is a typical behavioral variable in essence which integrates employee team-oriented behaviors with proficiency, adaptivity, and proactivity. It is established by the extant literature that regardless of the specific shaping basis, pay dispersion is more likely to at first incur individual employee’s psychological or emotional reactions and then affect his or her behaviors and performance (Heneman and Judge, 2000; Shaw, 2014). Thus, there may be distance between performance-based HPD and team member work role performance. This study has just revealed that, only through activating team member’s workplace benign envy could performance-based HPD play a positive role in achieving team member work role performance.

### Theoretical Implications

First, to our knowledge, this study is among the first to examine the relationships between performance-based HPD and team member work role performance, which is a timely response to many organizational practitioners’ concern as well as an attempt to bridge the research gap. In the ongoing uncertain, complex, and interdependent context where teams and pay-for-performance are in widespread adoption, many organizational practitioners are deeply concerned with the issue that performance-based HPD within a team could actually help organizations realize team member work role performance, which integrates employee team-oriented work role behaviors with proficiency, adaptivity, and proactivity as a team member in such a complex and dynamic context. However, almost no attention has been dedicated to this practically meaningful issue. Our study seeks to address it. We take horizontal pay comparisons and performance-shaping basis into consideration, which is distinct from prior studies that neither give nuance to pay comparison direction nor consider the specific shaping basis of pay dispersion (Becker and Huselid, 1992; Bloom, 1999; Aime et al., 2010; Uriesi, 2016), and explore why, when, and what effect performance-based HPD may exert on team member work role performance, which goes beyond previous studies that mainly concentrate on the employee outcome performance effects of pay dispersion. As such, we not only respond to many organizational practitioners’ main concern but also make efforts to plug the research gap.

Second, this study sheds new light on employees’ emotional reaction process that governs how they react to large performance-based HPD by identifying workplace benign envy as one of the most important emotional consequences of performance-based HPD and a key mediator transmitting the positive effect of performance-based HPD to team member work role performance. Specifically, this study proposes that when performance-based HPD is larger, a team member is more inclined to attribute a higher pay level as deservingness due to the legitimacy of its performance-shaping basis and tends to have a higher control potential perception because of the close performance-pay linkage, which thereby could give rise to team member’s workplace benign envy and ultimately may achieve team member work role performance due to the challenge-oriented actions within workplace benign envy. This theorizing not only enables us to identify workplace benign envy as one of the emotional consequences of performance-based HPD but also proposes a novel explanation (i.e., the workplace benign envy mechanism which goes beyond the explanation of Ensley et al. (2007) through team behavioral dynamics such as cohesion, conflict, and group potency) to the extant mechanism research of pay dispersion effects. Additionally, the significant role of workplace benign envy in transmitting the positive effect of performance-based HPD to team member work role performance suggests that pay dispersion effects research may benefit more from taking the legitimacy of specific shaping basis and employees’ psychological or emotional reactions into consideration, which opens a new but feasible and hopeful way to promote pay dispersion effects research.

Third, this study extends research on team member work role performance by identifying two important antecedents from the perspective of pay distribution characteristic (i.e., performance-based HPD) and employees’ emotional reactions toward pay dispersion (i.e., workplace benign envy). Over the past 30 years, in response to the growing uncertainty, complexity, and interdependence of work systems, a wide range of new constructs of employee performance such as citizenship performance (Smith et al., 1983), contextual performance (Borman and Motowidlo, 1993), adaptive performance (Hesketh and Neal, 1999), proactivity performance (Crant, 2000), and so on have been put forward. Based on them, Griffin et al. (2007) proposed a new but comprehensive view of employee performance in the ongoing dynamic context—work role performance, which thoroughly depicts work role behaviors that an employee should perform well as an individual, a team member, and an organization member. This study primarily focuses on team member work role performance due to the prevalent adoption of teams in organizations and confirms that performance-based HPD within a team and workplace benign envy are both important determinants. As such, it enriches the antecedent research of team member work role performance.

Finally, this study introduces a new perspective to identify the condition under which we could take full advantage of the mediating role of workplace benign envy in the relationship between performance-based HPD and team member work role performance. It suggests that a team member’s pay position plays an important moderating role in the mediating process of workplace benign envy, such that when a team member’s pay position is higher, large performance-based HPD tends to strengthen workplace benign envy, which thereby enhances team member work role performance. Thus, it identifies one important boundary condition of the mediating role of workplace benign envy in transmitting the positive effect of performance-based HPD to team member work role performance and offers a unique theoretical perspective on workplace benign envy by uncovering the interactive effect of performance-based HPD and pay position.

### Practical Implications

The findings of this study could provide significant practical implications for employee team-oriented behavioral performance management in the ongoing work systems.

First, organizations should utilize the indirect-positive role of performance-based HPD in achieving team member work role performance and take employee team-oriented behavioral performance such as work role behaviors with proficiency, adaptivity, and proactivity as a team member into consideration when establishing or adapting an employee performance appraisal system. HPD within a team *per se* merely represents unequal pay distribution among team members rather than pay inequity (Trevor et al., 2012), as the latter largely depends on the legitimacy of the specific shaping basis of HPD (Trevor and Wazeter, 2006; Aime et al., 2010). Since performance-based HPD could exert an indirect-positive effect on team member work role performance, organizations’ employee incentive plan within a team should consider the legitimacy of performance-shaping basis and make sure that HPD within a team is largely the result of performance difference which is of high legitimacy (Aime et al., 2010). Besides, since employee performance changes over time, performance within performance-based HPD should be updated and be anticipated to include employee team-oriented behavioral performance such as work role behaviors with proficiency, adaptivity, and proactivity as a team member. Surely, it is worthy to note that organizations should guarantee that employee performance appraisal is objective and impartial, because, if not, performance may be viewed as an unfair source of pay dispersion (Schwab, 1991).

Second, organizations should take full advantage of the mediation role of workplace benign envy in transmitting the positive effect of performance-based HPD to team member work role performance. This study confirms that only through activating team member’s workplace benign envy could performance-based HPD have a positive impact on team member work role performance, which demonstrates the positive influence of workplace envy. Therefore, it is unwise to blindly regard workplace envy as a taboo or a kind of shameful and socially condemned emotion (Vidaillet, 2007). In order to utilize the positive mediation role of workplace benign envy in the relationship between performance-based HPD and team member work role performance, organizational managers or team leaders should closely observe employees’ workplace envy, identify whether it is benign or malicious, and openly acknowledge, support, and encourage workplace benign envy. Moreover, organizations could activate or strengthen team member’s workplace benign envy through implementing a dispersed pay-for-performance plan within a team since performance-based HPD is positively related to team member’s workplace benign envy.

Finally, organizations should pay more attention to team members who are particularly inclined to engender workplace benign envy when performance-based HPD is large. Even if HPD is based on the legitimate performance-shaping basis, employees may not have the same emotional reactions toward HPD (Shaw, 2014). This study finds that the activating effect of performance-based HPD on workplace benign envy and the mediating role of workplace benign envy in the relationship between performance-based HPD and team member work role performance are much stronger when a team member is in a higher pay position, which helps organizations identify pay position as an important boundary condition of the activating effect of performance-based HPD on workplace benign envy and the mediating process of workplace benign envy. Therefore, when performance-based HPD is large, team members who are in a higher rather than a lower pay position are more likely to engender stronger workplace benign envy. If organizations want to efficiently activate employees’ benign workplace envy, they should pay greater attention to the management of employees in a higher pay position while implementing a dispersed pay-for-performance strategy.

### Limitations and Suggestions for Future Research

This study is subject to several limitations which should be noted now and addressed in the future. First, as the extant evidence of positive effects of pay dispersion is almost always based on samples that have clear and identifiable performance measures, such as sports team (Ehrenberg and Bognanno, 1990; Becker and Huselid, 1992; Gilsdorf and Sukhatme, 2008a, b), the conclusion that performance-based HPD indirectly promotes team member work role performance through activating team member’s workplace benign envy is mainly based on team samples with clear and identifiable performance measures such as sales workgroups, university faculty teams, and manufacturing departments. However, performance is essentially a multi-dimensional construct (Farkas and Anderson, 1979), and, not all kinds of employee performance could be identifiable (Shaw, 2014). Therefore, scholars should think about whether to introduce a variable that could depict the extent to which employee performance could be identifiable in the research model in the future, such as performance identifiability suggested by Shaw (2014), to further discuss its potential role in pay dispersion effects based on a variety of samples.

Second, although this study confirms that workplace benign envy completely mediates the relationship between performance-based HPD and team member work role performance since the regression coefficient of performance-based HPD (*X*) on team member work role performance (*Y*) becomes insignificant after the hypothesized mediator (i.e., workplace benign envy) entered the regression model (β = 0.026, *p* < 0.05 in Model 5→β = 0.014, *p* > 0.05 in Model 7 in Table 4) (Baron and Kenny, 1986), theoretically, other mediators may also play roles. For example, pay fairness, which is a well-established measure of employees’ psychological and emotional reactions toward pay dispersion (Trevor and Wazeter, 2006; Park et al., 2017), may be another mediator operating in parallel with workplace benign envy in transmitting the positive effect of performance-based HPD to team member work role performance. Hence, an important next step for future research is to explore the potential mediating roles of other mediators.

Third, besides team member’s pay position, other moderators may exist. The previous research has confirmed that some contextual factors and individual-level variables could influence workplace (benign/malicious) envy, such as differentiated LMX (Vecchio, 1995), individual’s core self-appraisal (Tai et al., 2012), self-esteem (Vecchio, 2005), and dispositional envy (Cohen-Charash and Mueller, 2007; Lange and Crusius, 2015; Pera, 2018). These factors may affect the indirect relationship between performance-based HPD and team member work role performance via workplace benign envy. For example, dispositional benign envy may enhance workplace benign envy and employee behavioral performance through person-environment fit in a large performance-based HPD situation. Therefore, the possible moderating roles of these factors in the mediating process of workplace benign envy should deserve special attention in future research.

Finally, the findings of this study are confirmed by ordinary employees who are working in Chinese organizational teams, which means that future research should conduct more empirical studies in the non-Chinese context to examine the external validity of findings.

## Data Availability Statement

The raw data supporting the conclusions of this article will be made available by the authors, without undue reservation.

## Ethics Statement

The studies involving human participants were reviewed and approved by the Human Research Ethics Committee (HREC) at School of Mathematics and Statistics, Xuzhou University of Technology. The patients/participants provided their written informed consent to participate in this study.

## Author Contributions

HZ contributed to all works including constructing the conceptual framework, collecting the empirical data, analyzing the data, writing the manuscript, and revising the manuscript. SS mainly contributed to collecting the empirical data, analyzing the data, writing the manuscript, and revising the manuscript. LZ mainly contributed to collecting empirical data. All authors contributed to the article and approved the submitted version.

## Conflict of Interest

The authors declare that the research was conducted in the absence of any commercial or financial relationships that could be construed as a potential conflict of interest.

## Appendix

### Workplace Benign Envy Scale

1. When I envy other team members who have a higher pay level, I mainly focus on how I could obtain the same or even a higher pay level in the future.
2. If I notice that my coworker is better than me, I will try my best to improve myself.
3. Envying other team members who have superiority in pay comparison motivates me to accomplish my goals.
4. I strive to reach my envied coworker’s excellent achievements.
5. If other team members have superior qualities, achievements, or possessions, I try my best to attain them for myself.

### Team Member Work Role Performance Scale

1. Team member A/B/C…can coordinate his or her work with other team members effectively.
2. Team member A/B/C…can communicate with other team members effectively.
3. Team member A/B/C…can provide help to other team members when asked, or needed.
4. Team member A/B/C…can deal with changes affecting his or her working unit effectively.
5. Team member A/B/C…can learn new skills or take on new roles to cope with changes in the way his or her unit works.
6. Team member A/B/C…can constructively respond to changes in the way his or her team works.
7. Team member A/B/C…can suggest ways to make his or her working unit more effective.
8. Team member A/B/C…can develop new methods to help his or her working unit perform better.
9. Team member A/B/C…can improve the way his or her working unit does things.
